# Fasting before or after wound injury accelerates wound healing through the activation of pro-angiogenic SMOC1 and SCG2

**DOI:** 10.7150/thno.44115

**Published:** 2020-02-19

**Authors:** Ming-Jie Luo, Shan-Shan Rao, Yi-Juan Tan, Hao Yin, Xiong-Ke Hu, Yan Zhang, Yi-Wei Liu, Tao Yue, Ling-Jiao Chen, Li Li, Ya-Rong Huang, Yu-Xuan Qian, Zheng-Zhao Liu, Jia Cao, Zhen-Xing Wang, Zhong-Wei Luo, Yi-Yi Wang, Kun Xia, Si-Yuan Tang, Chun-Yuan Chen, Hui Xie

**Affiliations:** 1 Department of Orthopedics, Xiangya Hospital, Central South University, Changsha, Hunan 410008, China; 2 Xiangya School of Nursing, Central South University, Changsha, Hunan 410013, China; 3 Movement System Injury and Repair Research Center, Xiangya Hospital, Central South University, Changsha, Hunan 410008, China; 4 School of Nursing, Xinjiang Medical University, Urumqi, Xinjiang 830000, China; 5 Department of Sports Medicine, Xiangya Hospital, Central South University, Changsha, Hunan 410008, China; 6 Hunan Key Laboratory of Organ Injury, Aging and Regenerative Medicine, Changsha, Hunan 410008, China; 7 Hunan Key Laboratory of Bone Joint Degeneration and Injury, Changsha, Hunan 410008, China; 8 National Clinical Research Center for Geriatric Disorders, Xiangya Hospital, Central South University, Changsha, Hunan 410008, China; 9 Department of Pathology, Sun Yat-sen Memorial Hospital, Sun Yat-sen University, Guangzhou, Guangdong 510220, China; 10 The First Affiliated Hospital, Xinjiang Medical University, Urumqi, Xinjiang 830000, China; 11 Changji Hospital of Traditional Chinese Medicine, Changji, Xinjiang 831100, China

**Keywords:** fasting, wound healing, angiogenesis, SMOC1, SCG2

## Abstract

Healing of the chronic diabetic ulceration and large burns remains a clinical challenge. Therapeutic fasting has been shown to improve health. Our study tested whether fasting facilitates diabetic and burn wound healing and explored the underlying mechanism.

**Methods:** The effects of fasting on diabetic and burn wound healing were evaluated by analyzing the rates of wound closure, re-epithelialization, scar formation, collagen deposition, skin cell proliferation and neovascularization using histological analyses and immunostaining. *In vitro* functional assays were conducted to assess fasting and refeeding on the angiogenic activities of endothelial cells. Transcriptome sequencing was employed to identify the differentially expressed genes in endothelial cells after fasting treatment and the role of the candidate genes in the fasting-induced promotion of angiogenesis was demonstrated.

**Results:** Two times of 24-h fasting in a week after but especially before wound injury efficiently induced faster wound closure, better epidermal and dermal regeneration, less scar formation and higher level of angiogenesis in mice with diabetic or burn wounds. *In vitro*, fasting alone by serum deprivation did not increase, but rather reduced the abilities of endothelial cell to proliferate, migrate and form vessel-like tubes. However, subsequent refeeding did not merely rescue, but further augmented the angiogenic activities of endothelial cells. Transcriptome sequencing revealed that fasting itself, but not the following refeeding, induced a prominent upregulation of a variety of pro-angiogenic genes, including SMOC1 (SPARC related modular calcium binding 1) and SCG2 (secretogranin II). Immunofluorescent staining confirmed the increase of SMOC1 and SCG2 expression in both diabetic and burn wounds after fasting treatment. When the expression of SMOC1 or SCG2 was down-regulated, the fasting/refeeding-induced pro-angiogenic effects were markedly attenuated.

**Conclusion:** This study suggests that fasting combined with refeeding, but not fasting solely, enhance endothelial angiogenesis through the activation of SMOC1 and SCG2, thus facilitating neovascularization and rapid wound healing.

## Introduction

Healing of the large burn wounds and chronic ulceration is still a therapeutic challenge 1. A variety of disease conditions such as diabetes mellitus negatively affect wound repair 2 and insufficient blood supply due to impaired angiogenesis represents a critical contributor to the failure to heal 3, 4. New vessels formed by pre-existing endothelial cells (angiogenesis) transport oxygen and nutrients to the wound sites, enabling keratinocytes and fibroblasts to grow, proliferate, migrate, generate new epidermis or synthesize collagen, which finally result in the healing of wound 1. Hence, numerous studies have been undertaken to explore the optimal strategies for rapid wound healing by inducing therapeutic angiogenesis.

The capacity to survive starvation is an adaptive response of animals and humans. Fasting is a practice that restricts the consumption of food or drink for certain times. Multiple forms of therapeutic fasting have been reported regarding their efficacy to improve health (decreasing body fat and blood pressure, promoting stem cell function and regeneration, reversing immunosuppression, suppressing inflammation, *etc.*), delay aging and extend life span 5-9. Recently, Xin *et al.* showed that fasting for 48 h during a 4-day observation period after stroke was able to augment angiogenesis in ischemic brain and alleviate cerebral ischemic injury in mice; periodic fasting (a 48-h period of fasting per week for one month) resulted in reduced cortical atrophy and long-term neurobehavioral improvement 10. Nonetheless, the regulatory molecular mechanism through which fasting affects angiogenesis remains unclear. This report, along with evidences that serum reduction increased the expression of angiogenesis-related genes in cultured human adipose stromal cells 11, 12, prompted us to test whether fasting facilitates wound repair and regeneration by stimulating angiogenesis and to investigate the mechanism underlying this process.

Here, we generated full-thickness excisional or burn skin wounds in streptozotocin-induced diabetic mice and normal mice, respectively, and determined whether fasting prior to or after wound injury for a certain period of time can promote angiogenesis and speed up the process of wound healing. *In vitro*, we evaluated the effects of fasting and refeeding on the proliferation, migration and angiogenic tube formation of endothelial cells. To further explore the molecular mechanism, transcriptome sequencing of fasting and non-fasting endothelial cells was conducted to screen the differentially expressed angiogenesis-related genes and the role of the candidate genes in the fasting-induced promotion of angiogenesis was demonstrated.

## Results

### Fasting before or after wound injury facilitates the healing of diabetic and burn wounds

**Figure 1:**
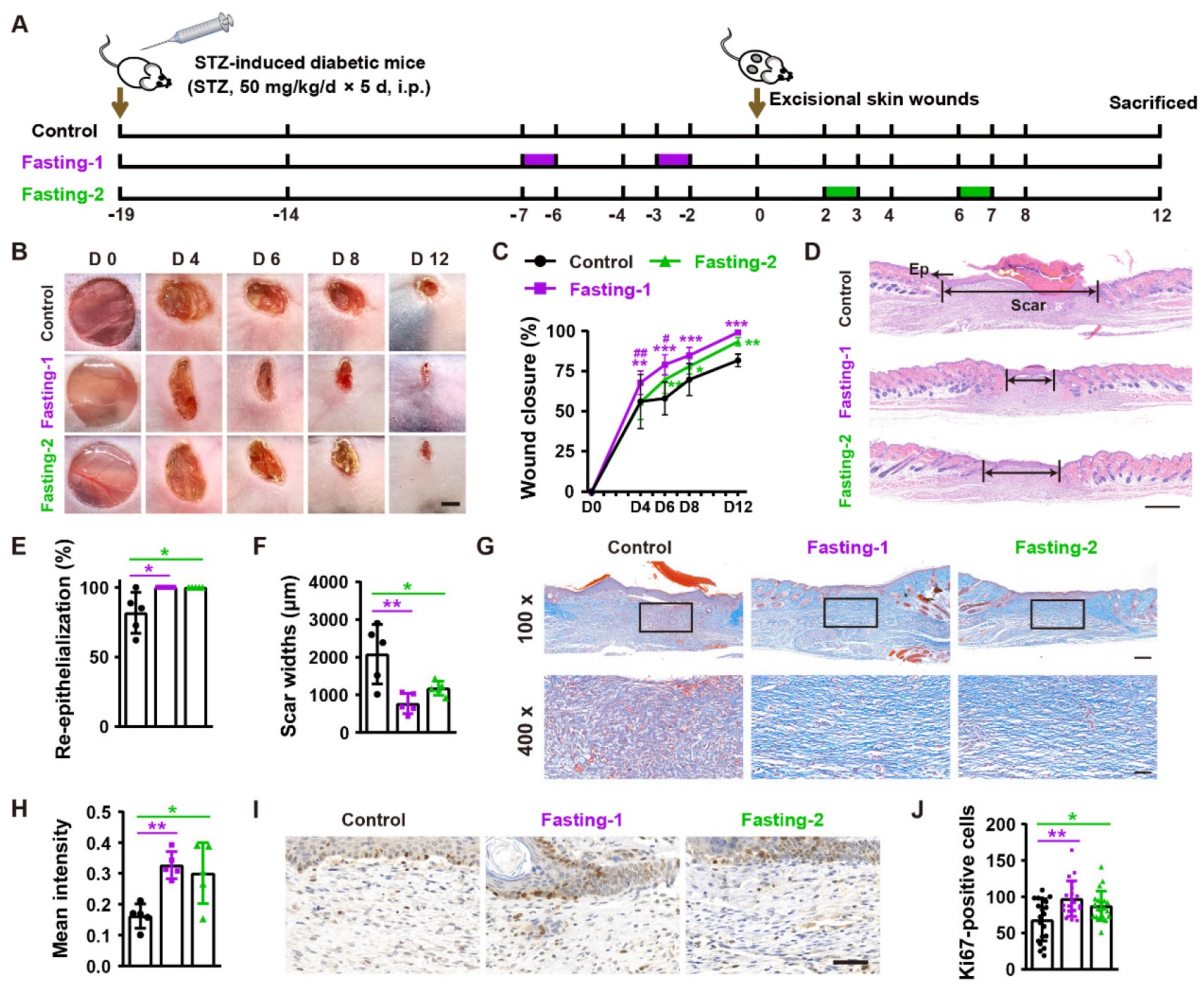
** Fasting before or after wound injury facilitates the healing of diabetic wounds. (A)** Illustration of the experimental procedure for testing the effects of fasting-1 and fasting-2 regimens on diabetic wound healing. STZ: streptozotocin. i.p.: intraperitoneal injection. **(B)** Gross images of diabetic wounds from mice subjected to fasting-1, fasting-2 or non-fasting (control) treatments at days 4, 6, 8 and 12 post-wounding. Scale bar: 2 mm. **(C)** Wound closure rates at the indicated time points.* n* = 10 *per* group. **(D)** H&E staining of diabetic wounds at days 12 post-wounding. The single- and double-headed arrows indicate the epithelium (Ep) and edges of scars, respectively. Scale bar: 500 μm. **(E** and** F)** The rates of re-epithelialization** (E)** and widths of scars** (F)**. *n* = 5 *per* group. **(G)** Masson's trichrome staining of diabetic wounds at days 12 post-wounding. Scale bar: 200 μm (top) or 50 μm (bottom). **(H)** The mean intensities for Masson-positive areas. *n* = 5 *per* group. **(I** and **J)** Ki67 staining images of diabetic wounds at days 12 post-wounding** (I)** and the numbers of ki67-positive skin cells** (J)**. Scale bar: 50 μm. *n* = 5 *per* group. For **(C)**: Two-way ANOVA combined with Bonferroni *post hoc* test. For **(E**, **F**,** H** and** J)**: One-way ANOVA combined with Bonferroni *post* hoc test. For **(C)**: *P < 0.05 vs. control group, #*P* < 0.05 vs. fasting-1 group. For **(C, E, F, H** and **J)**: *P < 0.05, **P < 0.01, ***P < 0.001.

To assess the effects of fasting on diabetic wound healing, we generated streptozotocin-induced diabetic mice and designed two forms of fasting (Fasting-1: mice received two times of 24-h fasting in a week (at days 2 and 6) before the generation of excisional wounds, without fasting treatment during and after skin injuries; Fasting-2: mice were fasted for 24 h at days 2 and 6 post-wounding, respectively) to treat these mice (**Figure 1A**). As indicated by the representative images of wounds at days 4, 6, 8 and 12 post-wounding in **Figure 1B** and quantification of the wound closure rates in **Figure 1C**, the diabetic mice in fasting-1 treatment group showed profoundly enhanced wound closure at all tested time points than *ad libitum*-fed control mice. Fasting-2 regimen did not induce rapid wound closure at the early phase, as revealed by the comparable closure rates at days 4 post-wounding between fasting-2 and control group. However, refeeding for 3 days after fasting caused significantly accelerated wound closure, thus leading to much higher closure rates in fasting-2 group at days 6 post-wounding than that in control group. After fasting for 24 h from days 6 post-wounding and then refeeding for 5 days, the closure rates in fasting-2 group were close to, but still lower that of fasting-1 group, suggesting the superiority of fasting-1 regimen. Hematoxylin and eosin (H&E) staining showed that both of these two forms of fasting regimens caused completely re-epithelialization of these diabetic wounds at 12 days post-wounding, whereas large areas of several wounds in control mice were not covered with newly formed epidermis (**Figure 1D** and **1E**). The mice receiving fasting treatments, especially those treated with fasting-1, had much shorter scar tissues (with absence of cutaneous fat and hair follicles in dermis 13) compared with control mice (**Figure 1D** and **1F**). The wound tissues of fasting-treated mice also exhibited more regularly and densely arranged collagen fibers than that of *ad libitum*-fed control mice, as revealed by Masson's trichrome staining (**Figure 1G** and **1H**). Immunohistochemical staining for ki67 indicated much more proliferating cells in the wound sites of fasting-treated mice compared with control mice, but fasting-1 regimen induced a trend towards a higher level of skin cell proliferation (**Figure 1I** and **1J**).

**Figure 2:**
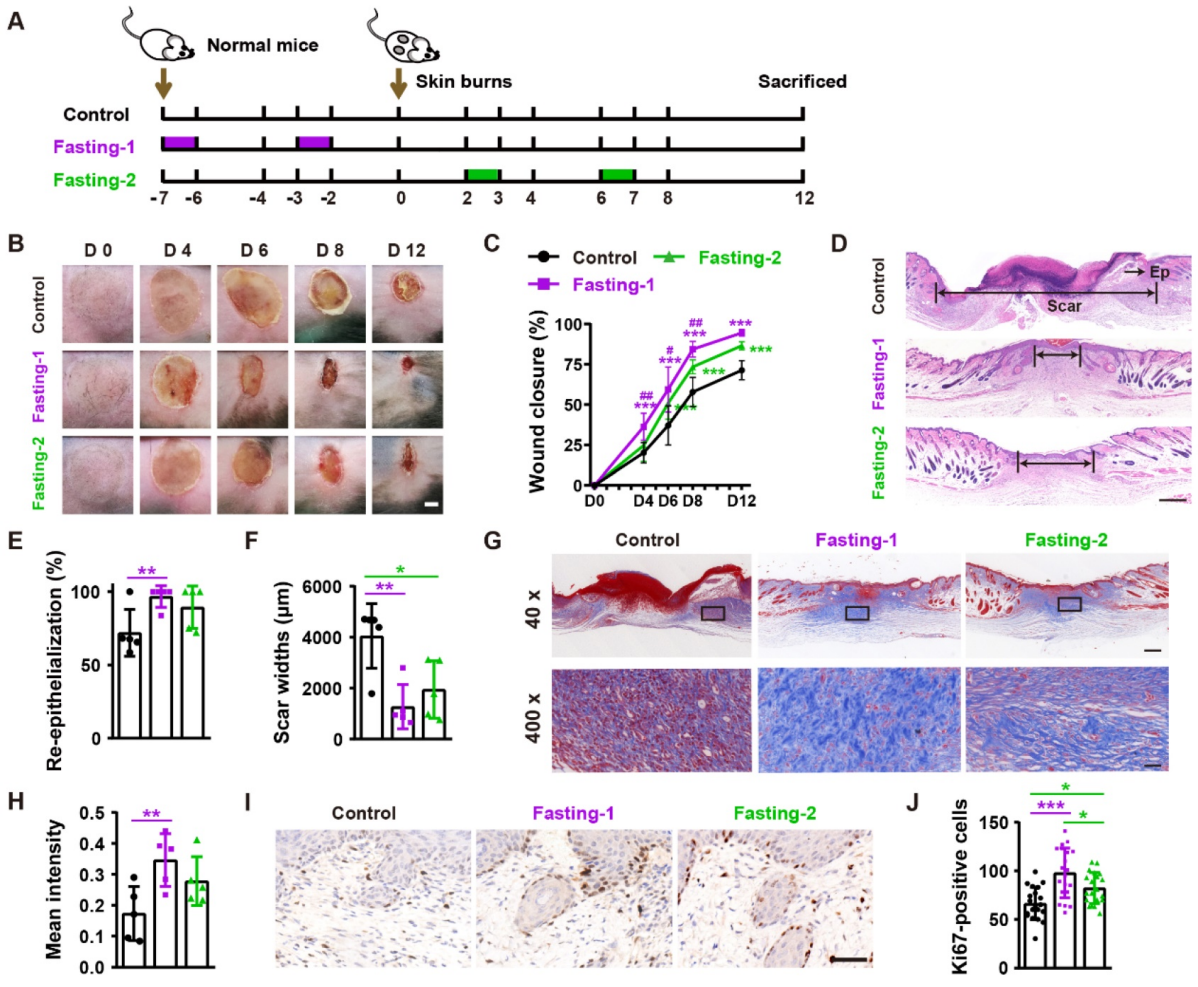
** Fasting before or after wound injury facilitates the healing of burns. (A)** Illustration of the experimental procedure for testing the effects of fasting-1 and fasting-2 regimens on burn wound healing.** (B)** Gross images of burn wounds from mice in different treatment groups at days 4, 6, 8 and 12 post-wounding. Scale bar: 2 mm. **(C)** The rates of wound closure rates.* n* = 10 *per* group. **(D)** H&E staining of burn wounds at days 12 post-wounding. Ep: epithelium. Scale bar: 500 μm. **(E** and** F)** The rates of re-epithelialization** (E)** and widths of scars** (F)**. *n* = 5 *per* group. **(G** and** H)** Masson's trichrome staining images of burn wounds at days 12 post-wounding** (G)** and the mean intensities for Masson-positive areas **(H)**. Scale bar: 500 μm (top) or 50 μm (bottom). *n* = 5 *per* group. **(I** and **J)** Ki67 staining images of burn wounds at days 12 post-wounding** (I)** and the numbers of ki67-positive skin cells **(J)**. Scale bar: 50 μm. *n* = 5 *per* group. For **(C)**: Two-way ANOVA combined with Bonferroni *post hoc* test. For **(E**,** F**,** H** and** J)**: One-way ANOVA combined with Bonferroni *post hoc* test. For **(C)**: *P < 0.05 vs. control group, #*P* < 0.05 vs. fasting-1 group. For **(C, E, F, H** and **J)**: *P < 0.05, **P < 0.01, ***P < 0.001.

We next tested the effects of fasting-1 (mice received two times of 24-h fasting in a week before burn injury) and fasting-2 (mice received two times of 24-h fasting at days 2 and 6 after burn injury) regimens on burn wound healing (**Figure 2A**). As shown in **Figure 2B** and **2C**, both fasting-1 and fasting-2 significantly induced rapid closure of burn wounds compared with control group. However, the positive effects of fasting-1 on wound closure at days 4, 6 and 8 post-wounding was much more remarkable that that of fasting-2 (**Figure 2B** and **2C**). Consistently, fasting-1 induced stronger effects than fasting-2 to induce higher extents of re-epithelialization and dermis regeneration (**Figure 2D-F**), higher levels of collagen deposition (**Figure 2G** and **2H**), and greater numbers of proliferating cells (**Figure 1I** and **1J**).

Together, these findings suggest that fasting no matter prior to or after wound injury is capable of accelerating wound repair and regeneration. Nevertheless, fasting before wounding is likely to be more beneficial to wound healing than fasting after wounding.

### Fasting before or after wound injury increases angiogenesis in the wound areas

**Figure 3:**
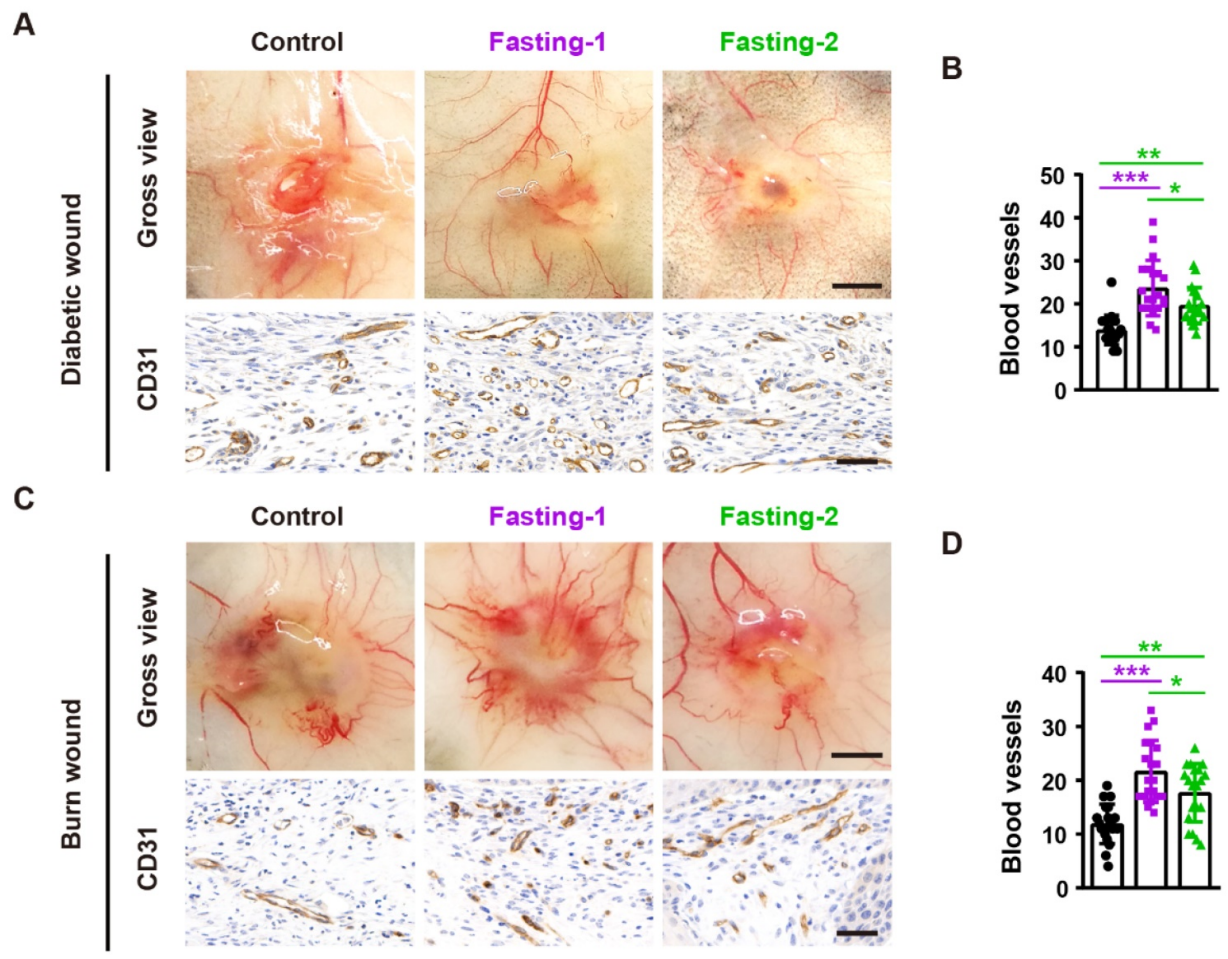
** Fasting before or after wound injury increases angiogenesis in the wound areas. (A)** Gross images from the undersurface (Scale bar: 2 mm) and CD31 staining images (Scale bar: 50 μm) of diabetic wounds from mice in different treatment groups at days 12 post-wounding. **(B)** The numbers of CD31-positive blood vessels in diabetic wounds. *n* = 5 *per* group. **(C)** Gross images from the undersurface (Scale bar: 2 mm) and CD31 staining images (Scale bar: 50 μm) of burn wounds at days 12 post-wounding.** (D)** The numbers of CD31-positive blood vessels in burn wounds. *n* = 5 *per* group. For **(B** and** D)**: One-way ANOVA combined with Bonferroni *post hoc* test. *P < 0.05, **P < 0.01, ***P < 0.001.

To determine whether fasting treatment promotes angiogenesis in the wound sites, we photographed the skin from the undersurface and conducted immunochemical staining for the wound tissues with the endothelial marker CD31. As shown in **Figure 3A** and **3B**, both fasting-1 and fasting-2 regimens enhanced the formation of blood vessels in the diabetic wounds and the numbers of CD31-positive vessels in fasting-1 group were much higher than that of fasting-2 group. Consistently, fasting-1 regimen also caused much more potent effects than fasting-2 approach to stimulate blood vessel formation in the burn wounds (**Figure 3C** and** 3D**). These findings suggest that the higher ability to activate angiogenesis may contribute to the superiority of fasting-1 than fasting-2 in the stimulation of wound healing.

### Fasting and refeeding augment the angiogenic activities of endothelial cells

**Figure 4:**
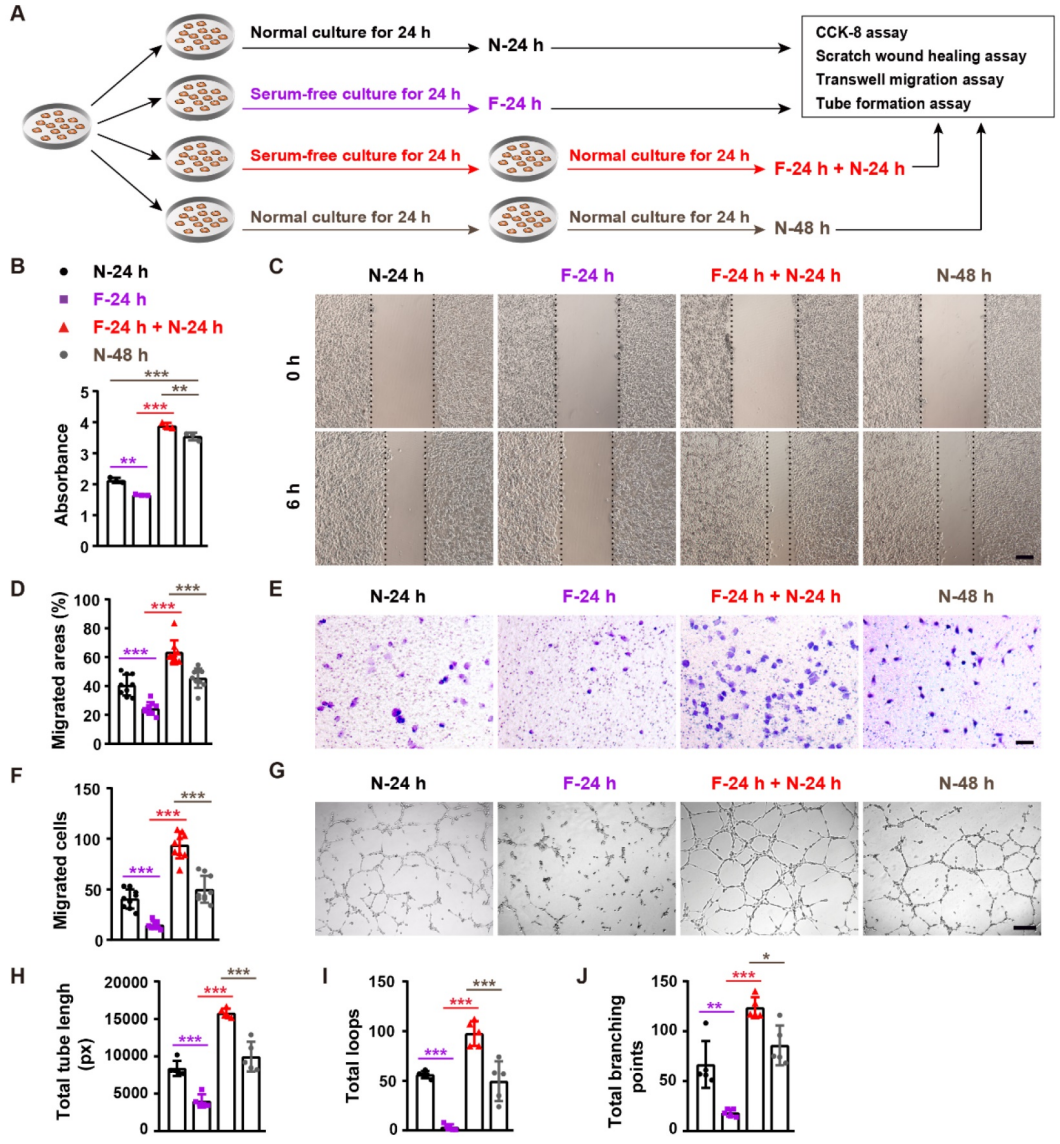
** Fasting and refeeding augment the angiogenic activities of endothelial cells. (A)** Schematic illustration of the experimental procedure for testing the effects of fasting/refeeding on endothelial angiogenesis. **(B)** CCK-8 analysis of the proliferation of endothelial cells subjected to fasting by serum deprivation for 24 h (F-24 h), fasting for 24 h and then refeeding in normal serum-containing medium for 24 h (F-24 h + N-24 h), or non-fasting (cultured in normal serum-containing medium) for 24 h (N-24 h) or 48 h (N-48 h). *n* = 3 *per* group. **(C** and** D)** Representative images of scratch wound healing assay of endothelial cells in different treatment groups **(C)** and the percentages of migration areas** (D)**. Scale bar: 250 μm. *n* = 3 *per* group. **(E** and** F)** Representative images of transwell migration assay **(E)** and the numbers of crystal violet-stained migrated cells **(F)**. Scale bar: 100 μm. *n* = 3 *per* group. **(G-J)** Representative tube formation images **(G)** and quantification of total tube length **(H)**, loops **(I)** and branching points **(J)**. Scale bar: 250 μm. *n* = 5 *per* group. For **(B**,** D**,** F**, **H**, **I** and** J)**: One-way ANOVA combined with Bonferroni *post hoc* test. *P < 0.05, **P < 0.01, ***P < 0.001.

We next used an *in vitro* serum deprivation cell model (mimicking the fasting-induced condition *in vivo*) to explore the direct effect of fasting on angiogenesis. As we found that refeeding after fasting, but not fasting alone, led to wound closure acceleration in mice, we thus not only assessed the effect of fasting, but also evaluated the impact of refeeding after fasting on the angiogenic activities of endothelial cells. After fasting by serum deprivation or non-fasting for 24 h, human microvascular endothelial cells (HMECs) were subjected to cell proliferation assay by cell counting kit-8 (CCK-8) analysis, scratch wound healing assay, transwell migration assay, and tube formation assay on Matrigel, or incubated for another 24 h in serum-containing growth medium and then subjected to these angiogenesis-related assays (**Figure 4A**).

CCK-8 assay revealed that fasting for 24 h (F-24h) resulted a significant reduction of HEMCs' proliferation compared with the cells incubated in normal serum-containing medium for 24 h (N-24 h) (**Figure 4B**). However, once the 24-h fasting cells were refed with serum-containing medium for 24 h (F-24 h + N-24 h), their proliferation was profoundly higher than that of the control cells treated with serum-containing medium for 48 h (N-48 h) (**Figure 4B**). Fasting/refeeding treatment also induced similar beneficial effects on the proliferation of mouse epidermal cell line JB6 and embryonic fibroblast NIH3T3 (**Supplementary Figure 1A** and **1B**). Scratch wound healing assay showed that the migration of HMECs in F-24 h was also lower than that of N-24 h group, but HMECs' migration in F-24 h + N-24 h group was much higher compared with N-48 h group (**Figure 4C** and** 4D**). The positive effect of fasting followed by refeeding on HMECs' migration was further confirmed by transwell migration assay (**Figure 4E** and** 4F**). Consistently, fasting for 24 h reduced the ability of HMECs to from tubes on Matrigel, whereas large numbers of vessel-like structures were formed by HMECs subjected to fasting for 24 h and refeeding for 24 h, as revealed by the tube formation photographs and quantification of total tube length, loops and branching points (**Figure 4G-J**). These data indicate that fasting combined with refeeding, but not fasting solely, enhances the angiogenic activities of endothelial cells.

### Fasting stimulates the expression of angiogenic genes in endothelial cells

**Figure 5:**
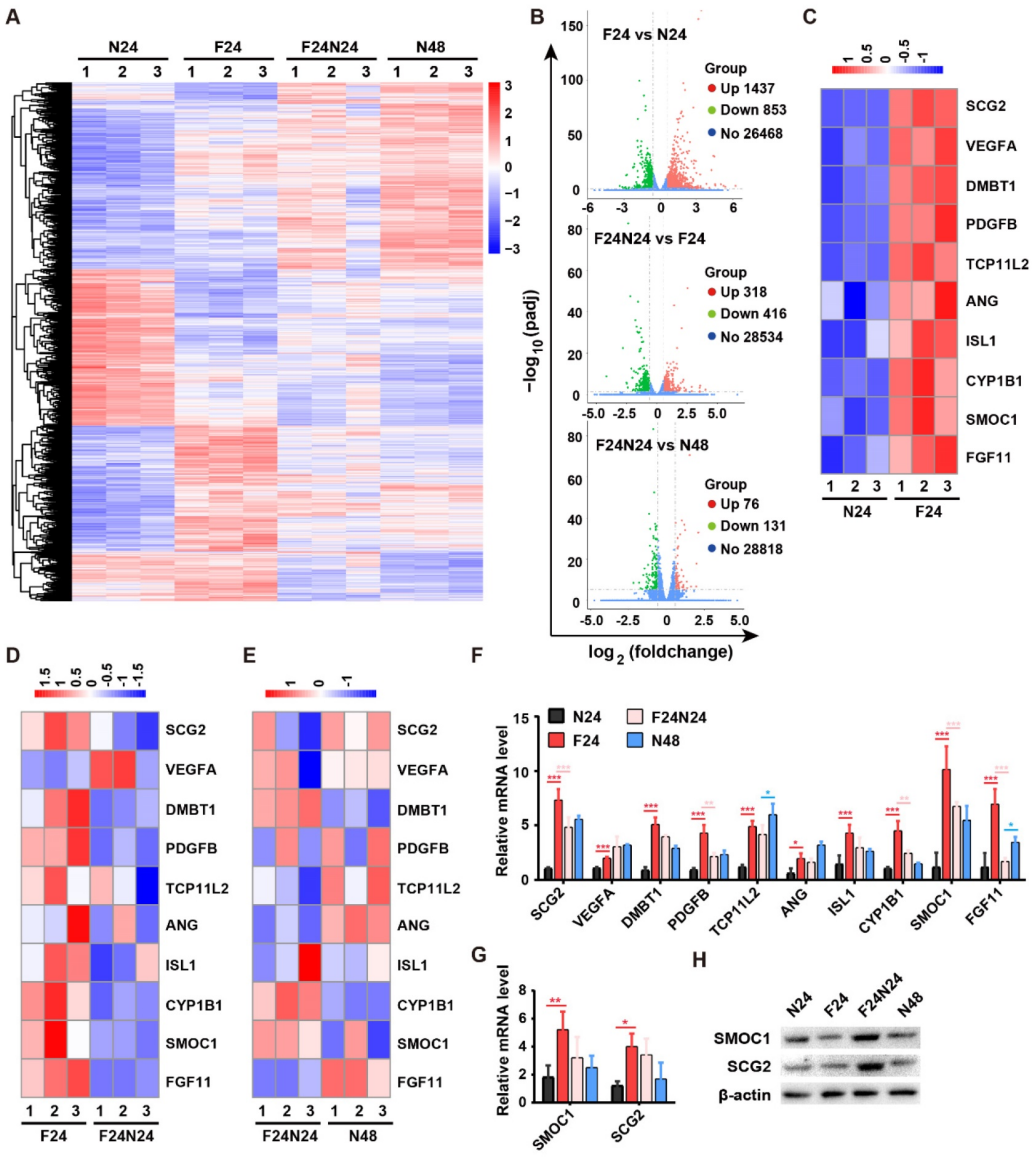
** Fasting stimulates the expression of angiogenic genes in endothelial cells. (A** and **B)** The differentially expressed genes (DEGs) in endothelial cells subjected to fasting alone for 24 h (F-24 h: F24), fasting for 24 h followed by refeeding for 24 h (F-24 h + N-24 h: F24N24), or non-fasting for 24 h (N-24 h: N24) or 48 h (N-48 h: N48) were illustrated as a heatmap **(A)** or three volcano plots **(B)**. |log_2_(foldchange)| > 0.58 and adjusted *p* value (padj) < 0.05 were used as the cutoff to identify DEGs. **(C-E)** Heatmaps showing the expression of a class of pro-angiogenic genes in different groups. **(F)** The expression of pro-angiogenic genes of interest was shown as a column diagram. *n* = 3 *per* group. **(G)** qRT-PCR analysis of the expression of *SMOC1* and *SCG2*. *n* = 3 *per* group.** (H)** Western blotting for SMOC1 and SCG2 in N24, F24, F24N24 and N48 groups. For **(F** and** G)**: One-way ANOVA combined with Bonferroni *post hoc* test. *P < 0.05, **P < 0.01, ***P < 0.001.

To explore the underlying molecular mechanism by which fasting/refeeding stimulates endothelial angiogenesis, we conducted transcriptome sequencing of HMECs in N-24 h (N24), F-24 h (F24), N-48 h (N48) and F-24 h + N-24 h (F24N24) groups to identify the potential molecules that mediate the pro-angiogenic action of fasting/refeeding. Differentially expressed genes (DEGs) with the cutoff of |log_2_(foldchange)| > 0.58 and adjusted *p* value (padj) < 0.05 were shown in **Supplementary Table 1** and illustrated as a heatmap in **Figure 5A** and as three volcano plots in **Figure 5B**. In total, 2290 genes were differently expressed between F-24 h and N-24 h groups, among which 1437 genes were up-regulated and 853 genes were down-regulated. Refeeding for 24 h after 24-h fasting induced the increased expression of 318 genes and decreased expression of 416 genes compared with F-24 h group, which resulted in the reduced gene expression differences between F-24 h + N-24 h and N-48 h groups. The DEGs were biologically interpreted and a number of pro-angiogenic genes were found to be up-regulated after fasting for 24 h, such as SPARC related modular calcium binding 1 (*SMOC1*), secretogranin II (*SCG2*), vascular endothelial growth factor A (*VEGFA*), deleted in malignant brain tumors 1 (*DMBT1*), platelet derived growth factor subunit B (*PDGFB*), angiogenin (*ANG*), cytochrome P450 family 1 subfamily B member 1 (*CYP1B1*), fibroblast growth factor 11 (*FGF11*), etc (**Figure 5C**). Refeeding for 24 h after 24-h fasting did not induce further increases, but resulted in decreases of the expression of these pro-angiogenic genes compared with F-24 h group, except *VEGFA* (**Figure 5D**). When the cells were maintained in normal serum-containing medium for 48 h (N-48 h group), the levels of these pro-angiogenic genes were also increased compared with N-24 h group, which led to similar, but not the same, gene expression profiles in F-24 h + N-24 h and N-48 h groups (**Figure 5E**). These data, which were also shown as a column diagram in **Figure 5F**, seemed to be paradoxical to the reduced angiogenic activities of endothelial cells in F-24 h group relative to N-24 h group and increased angiogenic abilities of endothelial cells in F-24 h + N-24 h group compared with N-48 h group. We hypothesized that fasting treatment might just evoke the angiogenic potential of endothelial cells, whereas the augmentation of endothelial angiogenesis indeed requires the refeeding process to provide enough nutrients for further protein translation and functional improvement.

We selected *SMOC1* and *SCG2*, two pro-angiogenic genes showing the highest and the second highest extent of up-regulation in response to fasting treatment, for further investigation. The up-regulation of *SMOC1* and *SCG2* after fasting was further determined by quantitative real-time PCR (qRT-PCR; **Figure 5G**). Western blotting for SMOC1 and SCG2 confirmed our hypothesis, which showed that fasting did not obviously affect or even decrease the protein levels of *SMOC1* or *SCG2* in HMECs, whereas refeeding after fasting induced prominent increases in the expression of these proteins (**Figure 5H**).

### SMOC1 and SCG2 partially mediate the fasting/refeeding-induced promotion of angiogenesis

**Figure 6:**
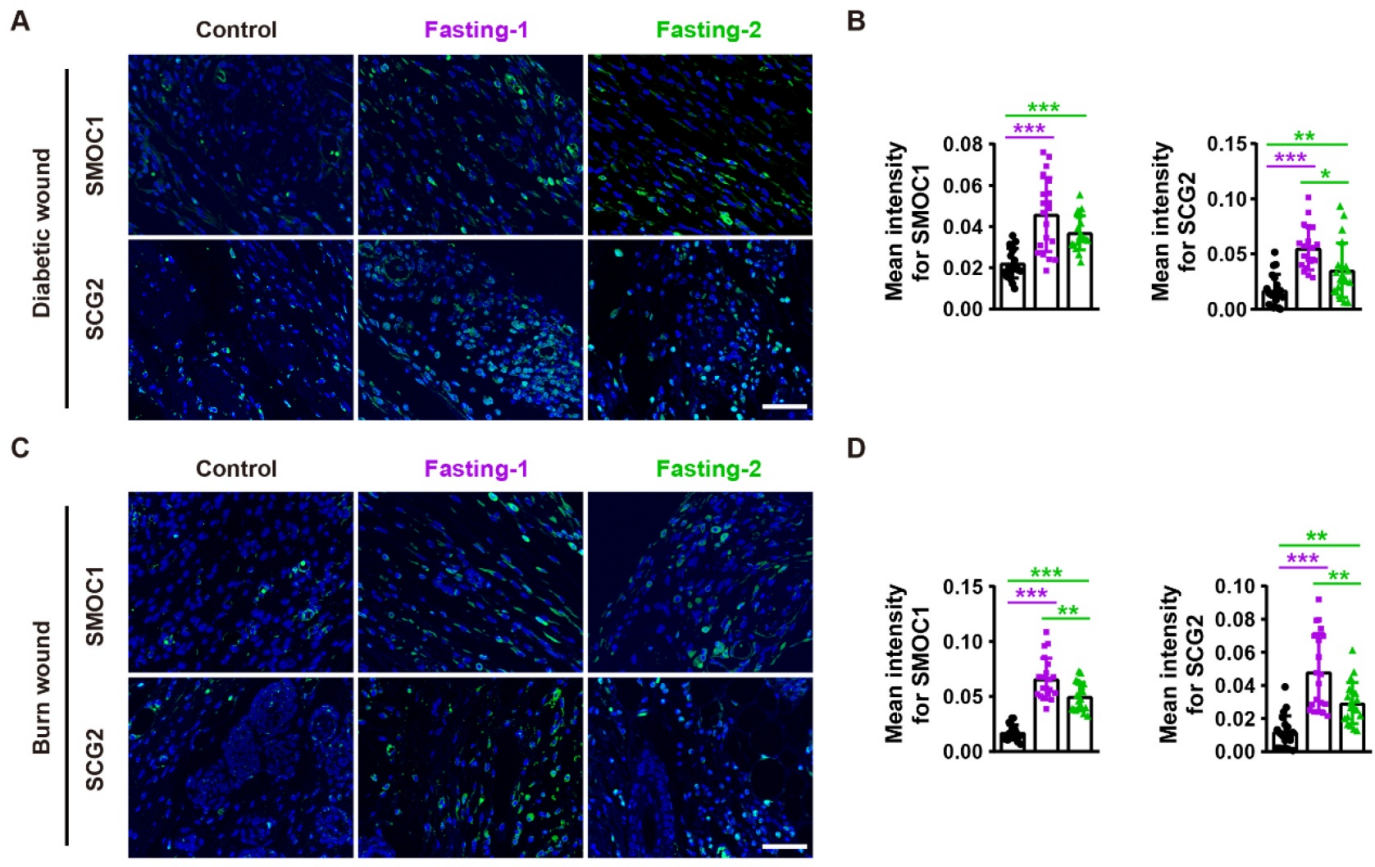
** Fasting before or after wound injury promotes SMOC1 and SCG2 expression in the wound areas. (A** and** B)** Immunofluorescent staining for SMOC1 and SCG2 in diabetic wounds at days 12 post-wounding **(A)** and the mean intensities for SMOC1- or SCG2-positive areas **(B)**. Scale bar: 50 μm. *n* = 5 *per* group. **(C** and** D)** Immunofluorescent staining for SMOC1 and SCG2 in burn wounds at days 12 post-wounding **(C)** and the mean intensities for SMOC1- or SCG2-positive areas **(D)**. Scale bar: 50 μm. *n* = 5 *per* group. For **(B** and** D)**: One-way ANOVA combined with Bonferroni *post hoc* test. *P < 0.05, **P < 0.01, ***P < 0.001.

We next asked whether SMOC1 and SCG2 contribute to the fasting/refeeding-induced stimulatory effects on angiogenesis. Immunofluorescent staining for SMOC1 and SCG2 revealed that both fasting-1 and fasting-2 regimens resulted in increases in the expression of these proteins in diabetic and burn wounds (**Figure 6A-D**), suggesting the involvement of these two proteins in the fasting/refeeding-induced promotion of angiogenesis* in vivo*.

**Figure 7:**
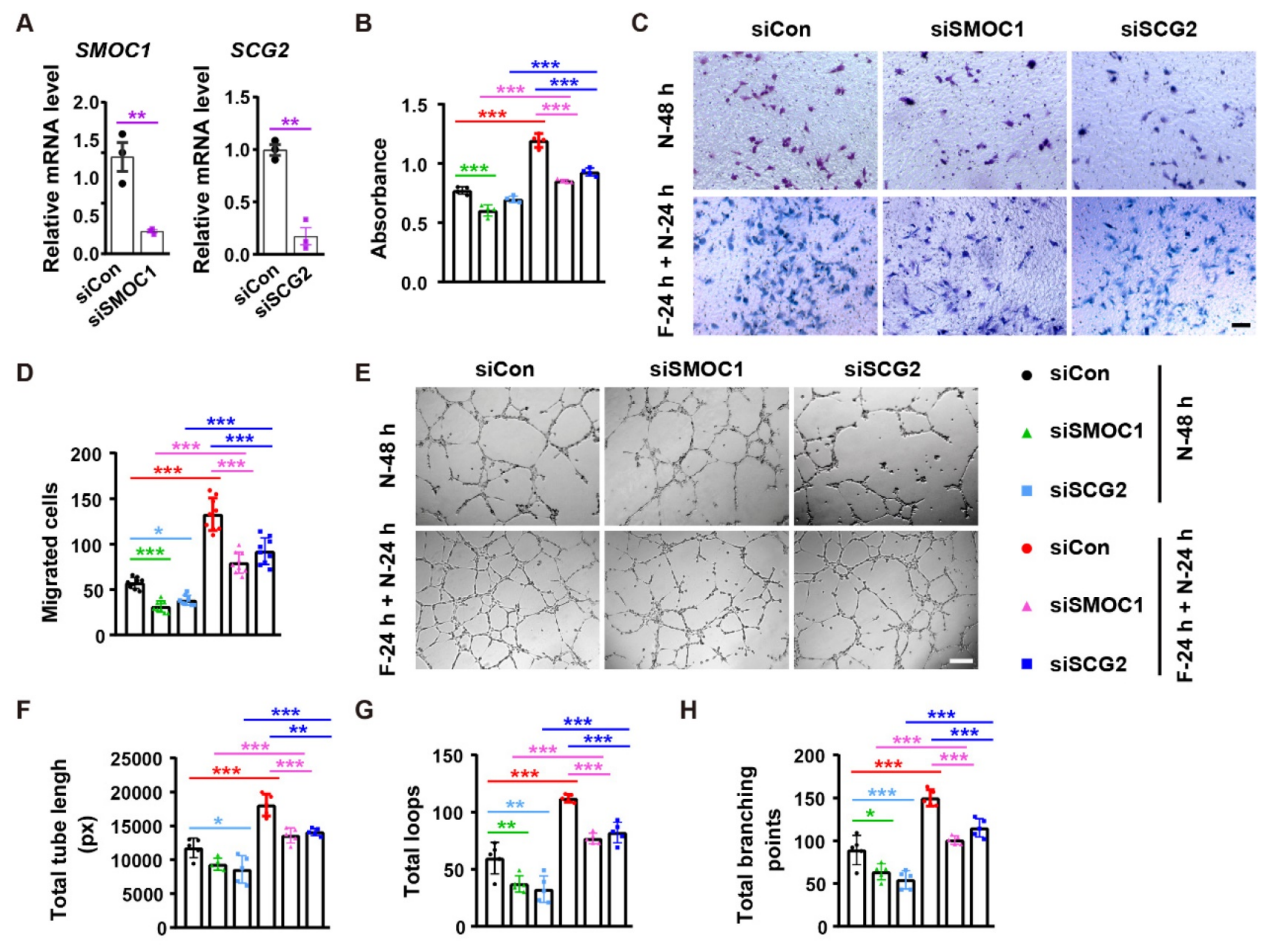
** SMOC1 and SCG2 partially mediate the fasting/refeeding-induced promotion of angiogenesis. (A)** qRT-PCR analysis of the expression of *SMOC1* and *SCG2*. *n* = 3 *per* group. **(B)** Endothelial cell proliferation was assessed by CCK-8 analysis. *n* = 4 *per* group. **(C** and** D)** Representative images of transwell migration assay **(C)** and the migrated cell numbers **(D)**. Scale bar: 100 μm. *n* = 3 *per* group. **(E-H)** Representative tube formation images **(E)** and quantification of total tube length **(F)**, loops **(G)** and branching points **(H)**. Scale bar: 250 μm. *n* = 5 *per* group. For **(A)**: Unpaired, two tailed student's *t*-test. For **(B**,** D**,** F**, **G** and** H)**: One-way ANOVA combined with Bonferroni *post hoc* test. *P < 0.05, **P < 0.01, ***P < 0.001.

We then used the specific siRNAs to down-regulate the expression of *SMOC1* and *SCG2* in endothelial cells *in vitro*. qRT-PCR showed the inhibitory efficiency of siSMOC1 and siSCG2 in HMECs (**Figure 7A**). CCK-8 assay revealed that the inhibition of *SMOC1* resulted in growth reductions in both control cells (N-48 h) and fasting/refeeding cells (F-24 h + N-24 h), but the impact was much more remarkable in F-24 h + N-24 h group compared with N-48 h group (**Figure 7B**). The knockdown of *SCG2* just caused a trend of decrease in the proliferation of HMECs in N-48 h group, while the fasting/ refeeding-induced beneficial effect on HMECs' proliferation was markedly reduced with the suppression of *SCG2* (**Figure 7B**). Consistently, transwell migration assay indicated that the silencing of these two proteins especially *SMOC1* induced higher levels of inhibition of HMECs' migration in F-24 h + N-24 h group compared to N-48 h group (**Figure 7C** and** 7D**). The angiogenic tube formation abilities of HMECs in both N-48 h and F-24 h + N-24 h groups were profoundly repressed with the inhibition of *SMOC1* or *SCG2*, whereas the reduction seemed to be more obvious in the siSCG2-treated control cells and siSMOC1-treated fasting/refeeding cells, as shown by tube formation assay (**Figure 7E-H**). The positive effects of fasting/refeeding on HMECs' proliferation, migration and tube formation were not entirely abolished by inhibition of *SMOC1* or *SCG2* (**Figure 7B-H**). These results suggest that SMOC1 and SCG2 are not only required for maintaining normal angiogenesis, but also partially mediate the fasting/ refeeding-induced augmentation of angiogenic responses of endothelial cells.

## Discussion

It has long been appreciated that fasting or caloric restriction for a period of time has profound health benefits in diverse species. Our study provided the first evidence that two times of 24-h fasting in a week before or after wound injury was sufficient to induce faster wound closure, enhance re-epithelialization and dermal regeneration, and reduce scar formation in mice with diabetic or burn wounds. The promotion of angiogenic responses of adjacent resident endothelial cells in the wound bed might be an important factor contributing to the beneficial effects of these fasting regimens on wound healing, as the mice receiving fasting treatment showed a marked increase of neovascularization in the wound sites and the cultured endothelial cells possessed increased proliferative, migratory and vessel-like structure formation abilities after fasting/refeeding treatment. Our findings suggest an alluring prospect of fasting as a new strategy to induce therapeutic angiogenesis for wound therapy.

Although previous studies have reported the benefits of fasting or caloric restriction on the promotion of β-cell mass and insulin secretion 7, renewal and regeneration of hematopoietic stem cells 8 and intestinal stem cells 14, and rejuvenation of multiple organs such as liver, muscle and brain 15, few evidences have shown that fasting or caloric restriction alone can induce the regeneration or/and rejuvenation of a tissue or an organ. During fasting, the organisms minimize energy expenditure partly by rapidly decreasing the sizes of multiple organs/systems (liver, kidney, *etc*.) and the numbers of various kind of cells including blood cells and β-cells; once refeeding, a coordinated process occurs, which is able to induce high levels of cellular proliferation and tissue regeneration 7, 8, 15, 16.

Consistent with these findings, here we found that fasting after injury (fasting-2 regimen) did not expedite the closure of diabetic and burn wounds during the early phase of healing, but several days of refeeding induced much higher extent of wound repair in these mice compared with control group. This might be interpreted in part by the suppression of cellular proliferation and angiogenesis in the wound areas by temporal fasting, as *in vitro* observation showed that fasting alone could not stimulate, but rather repress the proliferation of skin cells (keratinocytes, fibroblasts, *etc*.) and angiogenic activities of endothelial cells, while subsequent refeeding did not merely rescue, but further significantly augment cellular proliferation and endothelial angiogenesis. The mice receiving two cycles of fasting/refeeding before injury (fasting-1 regimen) exhibited faster wound closure throughout the entire observation period and regenerated more blood vessels in the wounds than those subjected to fasting/refeeding after injury. This was likely due to that the regenerative responses of fasting-1 regimen-treated mice had been evoked by fasting/refeeding before wound injury and they did not require time to go through the fasting period-induced adaptive responses during the repair process. Our results suggest that a period of fasting no matter before or after wounding can enhance the regenerative responses of mice upon refeeding, but the outcomes are better when fasting is conducted in a healthy state (without injury). If clinically used, for people who have suffered an injury, they cannot adopt the fasting-1 regimen, but can use the fasting-2 regimen for rapid wound healing. However, for healthy people or people with other diseases and suitable for fasting, the alluring benefits of fasting-1 regimen suggest that proper fasting combined with refeeding may be capable of augmenting the regenerative capacities of their skin tissues, which will enable their skin heal more quickly after injury or surgery. That is to say, fasting/refeeding either after injury or when we have no injury may provide health benefits to us.

Researchers have previously reported that human adipose stromal cells incubated in medium with 2% fetal bovine serum (FBS) showed significantly altered expression of a lot of genes compare with the cells cultured in medium containing 10% FBS, among which the levels of a class of pro-angiogenic genes such as *VEGF*,* BMP6*, *FGF2*, *FGF9* and *IGF1* were profoundly increased in response to serum reduction 11, 12, suggesting that fasting may be able to augment the ability of adipose stromal cells to stimulate angiogenesis by favoring the secretion of pro-angiogenic factors. In this study, we demonstrated that fasting followed by refeeding, but not fasting alone, markedly enhanced the angiogenic activities of endothelial cells. It seemed to be that refeeding after fasting, but not fasting itself, activated the angiogenic potential of endothelial cells. However, transcriptome sequencing revealed that 24-h fasting by serum deprivation induced the remarkable changes of gene expression profiles in vascular endothelial cells, including significant increases in the expression of a variety of pro-angiogenic genes involved in endothelial cell proliferation, migration or/and capillary-like tube formation, such as *SMOC1* 17, *SCG2* 18, 19, *FGF11* 20, *DMBT1* 21, *PDGFB* 22,* etc*. Once refeeding for 24 h, the expression of most of the altered genes was restored to the levels similar to that of cells maintained in normal cultures throughout the 48-h observation period. These findings suggest that the angiogenic potential of endothelial cells is actually activated during the fasting period, whereas subsequent refeeding is essential for relieving the fasting-induced stressful conditions and really augmenting the angiogenic activities of endothelial cells.

The *SMOC1* gene encodes a secreted matricellular protein that is highly expressed in proliferating endothelial cells and promotes endothelial cell proliferation, migration, and tube formation partly by binding to endoglin, a type III auxiliary receptor for the transforming growth factor β (TGFβ) superfamily, and thereby altering the TGFβ signaling from activin receptor-like kinase (ALK5)/SMAD Family Member 2 (SMAD2) towards the activation of ALK1/SMAD1/5 17. SCG2 protein, a member of the chromogranin/secretogranin family, is rapidly cleaved into bioactive peptides in the tissues where it is generated. Among the cleaved products, secretoneurin has been shown to suppress endothelial cell apoptosis and enhance the proliferation, migration and angiogenesis of endothelial cells by stimulating VEGF signaling, MAPK system and/or PI3-kinase/Akt pathway 18, 19, 23. In this study, we not only found that the expression of *SMOC1* and *SCG2* at the mRNA levels was markedly increased in the cultured endothelial cells in response fasting, but also found that the levels of SMOC1 and SCG2 proteins were profoundly enhanced in skin tissues of the fasting-treated mice. When the expression of *SMOC1* and *SCG2* was down-regulated in endothelial cells, the fasting/refeeding-induced pro-angiogenic effects on endothelial cells were significantly blunted compared with control group. Our results suggest the necessary roles of SMOC1 and SCG2 in the fasting/refeeding-induced pro-angiogenic effects on endothelial cells, which may finally facilitate neovascularization and rapid wound repair and regeneration. A limitation in our study is that we did not assess the changes of the reported downstream factors of SMOC1 and SCG2 in either the cultured endothelial cells or the skin tissues after fasting/refeeding treatment.

Since other benefits regarding cycles of fasting and refeeding have been shown, such as promoting stem cell production 8, 14, reversing streptozotocin-induced β-cell depletion and reducing glucose level 7, and attenuating inflammation-associated skin lesions 15, fasting treatment may also act through other mechanisms to accelerate diabetic and burn wound healing, which still needs further investigation.

## Materials and Methods

### Animals

This study was approved by the Ethics Committee of Xiangya Hospital of Central South University. Two months old female C57BL/6 mice were used for animal experiments. To detect the effects of fasting on diabetic or burn wound healing, the mice were divided into three groups (*n* = 10 *per* group): 1) Control group: mice with diabetic/burn skin wounds received no treatments; 2) Fasting-1 group: diabetic or normal mice received two times of 24-h fasting in a week (at days 2 and 6) before the generation of diabetic or burn skin wounds; 3) Fasting-2 group: mice with diabetic/burn skin wounds were fasted for 24 h at 2 and 6 days post-wounding, respectively). When the mice in fasting-1 and fasting-2 groups were not fasting, they were fed *ad libitum* with a standard solid diet (Hunan SJA Laboratory Animal Co., Ltd., Changsha, Hunan), the same as that of the control mice. The models of diabetes were established by intraperitoneal injection of streptozotocin (50 mg/kg; Sigma-Aldrich, St. Louis MO, USA) daily for 5 days. One week later, 6-mm-diameter full-thickness excisional skin wounds were created on the diabetic mice. Burn models were created by placing a 1-cm-diameter metal rod (25 g; heated to 95-100°C in boiling water) vertically on the mouse back skin for 6 s without additional pressure. The procedures for generation of mouse models with diabetic or burn wounds were described in detail in our previous studies 1, 21. Images of wounds were obtained at days 0, 4, 6, 8 and 12 post-wounding. The rates of wound closure were analyzed as follows 1, 21:

wound closure (%) = (A_0_ - A_t_)/A_o_×100.

A_0_ represents the areas of wound at day 0, and A_t_ represents the areas of wound at other tested time points. The mice were killed at days 12 post-wounding. The wounds were harvested and images of wounds from the undersurface were obtained. The wound tissues were then processed for downstream analyses.

### Histological, immunohistochemical and immunofluorescent staining

The wound tissues were fixed for 24 h in 4% paraformaldehyde, dehydrated by graded ethanol, embedded in paraffin and sliced into 4-μm-thick sections. Re-epithelialization and scar formation were assessed by H&E staining. Collagen deposition was detected by Masson's trichrome staining. Cell proliferation and blood vessel formation were tested using immunohistochemical staining for ki67 and CD31, respectively. The procedures were conducted as previously described in our published study 1 by using the reagents purchased from Servicebio (Wuhan, Hubei, China). Anti-SMOC1 and anti-SCG2 used for immunofluorescent staining were purchased from Santa Cruz Biotechnology (Santa Cruz, California, USA). Images were obtained using an optical microscope (Olympus CX31; Tokyo, Japan). The rates of re-epithelialization, scar widths and mean intensity for Masson were quantified from H&E and Masson's trichrome staining images of five wound samples for each group. The numbers of Ki67^+^ proliferating cells and CD31^+^ blood vessels, and mean intensities for SMOC1^+^ or SCG2^+^ areas were quantified from four random visual fields of each wound section and five wound samples for each group.

### Cell culture and treatments

HMECs were normally grown in complete MCDB131 medium (Gibco, Grand Island, USA) containing 10% FBS (Gibco) and 1% GlutaMAX (Gibco). JB6 and NIH3T3 were normally cultured in complete high-glucose DMEM (Gibco) containing 10% FBS (Gibco). To assess the effects of fasting and refeeding on the angiogenic activities of HMECs and proliferation of JB6 and NIH3T3, these cells were divided into four groups: 1) N24 group: cells were cultured in complete MCDB131 or DMEM media for 24 h; 2) F24 group: cells were cultured in serum-free MCDB131 or DMEM media for 24 h; 3) F24N24 group: cells were cultured in serum-free MCDB131 or DMEM media for 24 h and then incubated in serum-containing complete MCDB131 or DMEM media for another 24 h; 4) N48 group: cell were cultured in complete MCDB131 or DMEM media for 48 h. Cultures were maintained at 37 °C in 5% CO_2_.

### CCK-8 assay

Cells (1 × 10^4^ cells *per* well) were plated into a 96-well culture plate and divided into different groups after incubation for 24 h. After receiving different treatments at the indicated times, the culture media were replaced by fresh media (100 μL *per* well) added with CCK-8 reagent (10 μL *per* well; 7Sea Biotech, Shanghai, China), followed by incubation of cells for 3 h at 37 °C. The absorbance at 450 nm was read by a microplate reader (Bio-Rad 680, Hercules, USA).

### Scratch wound healing assay

Cells from different groups (*n* = 3 *per* group) were plated into a 12-well plate at a density of 2 × 10^5^ cells *per* well. Once the cells were attached, a sterile p200 pipette tip was used to scratch the cell monolayer, followed by washing the cells with PBS. Cells were maintained in fresh complete media containing mitomycin-C (5 µg/mL; Sigma), which was used to avoid the interference of cellular proliferation. Images of cells at 0 h and 6 h post-wounding were obtained. The ratio of closure areas at 6 h post-wounding to wound areas at 0 h post-wounding was defined as the migration rate, which was analyzed from three random visual fields of wound areas for each group.

### Transwell migration assay

Cells from different treatment groups (*n* = 3 *per* group) were suspended in low-serum (5% FBS) medium at a density of 1 × 10^4^ cells *per* well and plated into the upper chamber of a 24-well plate with transwell inserts of 8 μm pore size (Corning, NY, USA). Complete medium (10% FBS; 500 μL *per* well) was added to the lower chamber and served as a chemoattractant. After overnight incubation, cotton swabs were used to remove the non-migrating cells on the upper surface of the filter. Cells on the lower surface of the filter (migrated cells) were stained with 0.5% crystal violet and then photographed. The number of migrated cells were quantified from three random visual fields of each group.

### Tube formation assay

Growth factor-reduced Matrigel (50 μL *per* well; BD Biosciences, San Jose, USA) was seeded into a 96-well plate and maintained at 37ºC for 0.5 h. Cells (2 × 10^4^ cells *per* well; n = 5 *per* group) from different treatment groups were plated into the Matrigel-coated wells and cultured at 37ºC for 6 h. Images of cells in each well were obtained and tube formation was evaluated by quantifying the total tube length, loops and branching points using the Image-Pro Plus 6.0 software.

### Gene expression profile analysis

RNA samples were processed for transcriptome sequencing and bioinformatics analysis by Novogene Bioinformatics Technology Co. Ltd (Beijing, China) to assess gene expression profiles in HMECs from different groups. Briefly, total RNA was extracted and the purity and integrity of RNA were assessed with a NanoPhotometer^®^ spectrophotometer (IMPLEN, CA, USA) and an Agilent 2100 Bioanalyzer (Agilent Technologies, CA, USA), respectively. Subsequently, mRNA was purified using poly-T oligo-conjugated magnetic beads, fragmented using divalent cations and reversely transcribed into cDNAs. After an end repair process and adenylation of 3' ends, the cDNA fragments were then ligated to the NEBNext Adaptor, followed by purification of the products with AMPure XP system. The fragments of preferentially 250~300 bp in length were enriched by PCR and the cDNA library was created. After quality assessment and cluster generation, transcriptome sequencing was conducted on an Illumina Hiseq 2000 platform that generated 125 bp/150 bp paired-end reads. After removing low-quality reads and reads containing adapter or ploy-N from raw data, the clean reads were obtained. Hisat2 and featureCounts v1.5.0-p3 were used for reads mapping to the reference genome and counting the numbers of mapped reads for each gene, respectively. Read counts were normalized to FPKM values. Differential expression analysis was conducted with DESeq2 R package (1.16.1) and genes with adjusted *P*-value (Padj) < 0.05 and |log_2_(foldchange)| > 0.58 were identified as DEGs between comparison groups 24. The Gene ontology (GO) database was used for the functional annotation of DEGs.

### RNA interference

To inhibit the expression of SMOC1 and SCG2 in HMECs, SMOC1 siRNAs (siSMOC1; RiboBio, Guangzhou, China) and siSCG2 (RiboBio) were used to interfere with HMECs, respectively. Cells treated with the universal negative control siRNAs (siCon) served as controls. After transfection for 24 h with riboFECT CP Transfection Kit (Invitrogen, Carlsbad, USA), the cells were harvested for qRT-PCR to confirm the inhibitory efficiency of these siRNAs. To assess the role of SMOC1 and SCG2 in the fasting/refeeding-induced regulation of endothelial angiogenesis, HMECs were transfected with siCon, siSMOC1 or siSCG2 in serum-containing media for 24 h and the culture media were then replaced with fresh serum-free or serum-containing media. After incubation for 24 h, these cells were then cultured in fresh serum-containing media for another 24 h and subjected to CCK-8 proliferation assay, transwell migration assay and tube formation assay as described above. The siRNA sequences were as follows: siSMOC1, 5'-GAGCAAGTGTCGCCTGGAG-3'; siSCG2, 5'-GCTTTGGAGTACATAGAAA-3'.

### qRT-PCR analysis

Total RNA was extracted from HMECs in different groups and cDNA was synthesized from 1 μg of total RNA using the All-in-One cDNA Synthesis SuperMix (Biotool, Houston, USA). qRT-PCR was carried out using FTC-3000 real-time PCR system (Funglyn Biotech Inc., Toronto, Canada) with SYBR Green qPCR Master Mix (Bimake, Houston, USA). GAPDH acted as an internal control and the relative gene expression was analyzed using the 2^-△△CT^ method. Primer sequences for qRT-PCR were as follows: *SMOC1*: forward, 5'-TCTATTCGTGTGACCAGGAGAG-3', and reverse, 5'-GGATGACAATACCCTCACGGG-3'; *SCG2*: forward, 5'-ACCAGACCTCAGGTTGGAAAA-3', and reverse, 5'-AAGTGGCTTTCATCGCCATTT-3'; *GAPDH*: forward, 5'-ATCCCATCACCATCTTCC-3', and reverse, 5'-GAGTCCTTCCACGATACCA-3'.

### Western blotting

Western blot analysis was conducted as described in our previous studies 25-27. Briefly, protein samples (30 μg) were separated by 10% SDS-PAGE and blotted onto PVDF membranes, followed by blocking for one hour in 5% milk. After that, the membranes were incubated overnight with antibodies targeting SMOC1 (Santa Cruz), SCG2 (Santa Cruz) or β-actin (Servicebio), and then immunoreacted with the secondary antibodies (Servicebio) after washing. An enhanced chemiluminescence kit (Advansta, Menlo Park, USA) was then used to detect the protein bands.

### Statistical analysis

Data were presented as mean ± SD. Two-group comparison was performed using unpaired, two tailed student's *t*-test. One-way analysis of variance (ANOVA) or two-way ANOVA with Bonferroni *post hoc* test was employed for multiple-group comparisons. *P* < 0.05 was statistically significant and indicated with * or ^#^; *P* < 0.01 was indicated with ** or ^##^; *P* < 0.001 was indicated with *** or ^###^.

## Abbreviations

H&E: Hematoxylin and eosin
HMECs: human microvascular endothelial cells
CCK-8: cell counting kit-8
DEGs: Differentially expressed genes
Padj: adjusted *p* value
SMOC1: SPARC related modular calcium binding 1
SCG2: secretogranin II
VEGFA: vascular endothelial growth factor A
DMBT1: deleted in malignant brain tumors 1
PDGFB: platelet derived growth factor subunit B
ANG: angiogenin
CYP1B1: cytochrome P450 family 1 subfamily B member 1
FGF11: fibroblast growth factor 11
qRT-PCR: quantitative real-time PCR
FBS: fetal bovine serum
TGFβ: transforming growth factor β (TGFβ)
ALK5: activin-like kinase 5
ALK1: activin-like kinase 1
SMAD1: SMAD Family Member 1
SMAD2: SMAD Family Member 2
SMAD5: SMAD Family Member 5
STZ: streptozotocin
GO: Gene ontology
