# Supplementary material for: Fasting before or after wound injury accelerates wound healing through the activation of pro-angiogenic SMOC1 and SCG2
Source: Theranostics. 2020 Feb 19;10(8):3779–92. doi: 10.7150/thno.44115 (PMC7069085; doi:10.7150/thno.44115)
Supplement: Supplementary file 1 — Supplementary figure. [file thnov10p3779s1.pdf]

Supplementary Material

Fasting before or after wound injury accelerates wound healing through the activation of pro-angiogenic SMOC1 and SCG2

Ming-Jie Luo[1-4#], Shan-Shan Rao[1-3#], Yi-Juan Tan[1,3], Hao Yin[1,3], Xiong-Ke Hu[1,3], Yan Zhang[3,5], Yi-Wei Liu[3,5], Tao Yue[1,3], Ling-Jiao Chen[9], Li Li[10], Ya-Rong Huang[11], Yu-Xuan Qian[1,3], Zheng-Zhao Liu[3,5,8], Jia Cao[1,3], Zhen-Xing Wang[1,3], Zhong-Wei Luo[1,3], Yi-Yi Wang[1,3], Kun Xia[1,3], Si-Yuan Tang[2*], Chun-Yuan Chen[1,3*], Hui Xie[1,3,5-8*]

[1]Department of Orthopedics, Xiangya Hospital, Central South University, Changsha, Hunan 410008, China

[2]Xiangya School of Nursing, Central South University, Changsha, Hunan 410013 China

[3]Movement System Injury and Repair Research Center, Xiangya Hospital, Central South University, Changsha, Hunan 410008, China

[4]School of Nursing, Xinjiang Medical University, Urumqi, Xinjiang 830000 China

[5]Department of Sports Medicine, Xiangya Hospital, Central South University, Changsha, Hunan 410008, China

[6]Hunan Key Laboratory of Organ Injury, Aging and Regenerative Medicine, Changsha, Hunan 410008 China

[7]Hunan Key Laboratory of Bone Joint Degeneration and Injury, Changsha, Hunan 410008, China

[8]National Clinical Research Center for Geriatric Disorders, Xiangya Hospital, Central South University, Changsha, Hunan 410008, China

[9]Department of Pathology, Sun Yat-sen Memorial Hospital, Sun Yat-sen University, Guangzhou, Guangdong, 510220, China

[10]The First Affiliated Hospital, Xinjiang Medical University, Urumqi, Xinjiang 830000 China

[11]Changji Hospital of Traditional Chinese Medicine, Changji, Xinjiang 831100 China

[#]These authors contributed equally to this work.

[*]Corresponding authors: Hui Xie (huixie@csu.edu.cn); Chun-Yuan Chen (chency19@csu.edu.cn); Si-Yuan Tang (sytang263@csu.edu.cn).

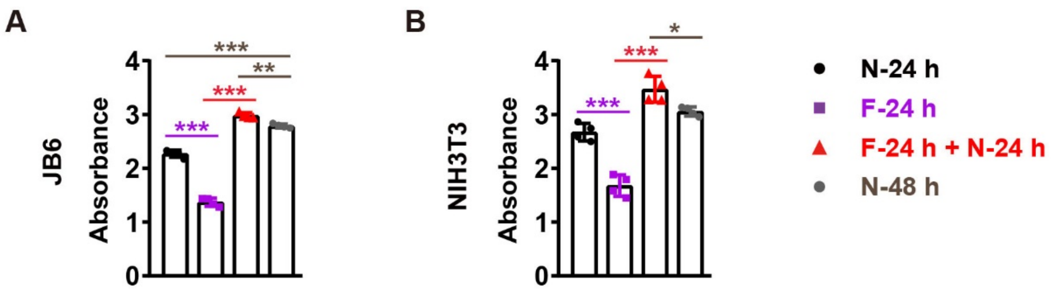

Supplementary Figure 1. The effects of fasting and refeeding on the proliferation of epidermal cells and fibroblasts. (**A** and **B)** CCK-8 analysis of the proliferation of mouse epidermal cell line JB6 **(A)** and embryonic fibroblast NIH3T3 **(B)** in different treatment groups. *n* = 4 *per* group. One-way ANOVA combined with Bonferroni *post hoc* test. *$P < 0.05$, **$P < 0.01$, ***$P < 0.001$.

Supplementary Table 1. DEGs in endothelial cells subjected to fasting/refeeding or non-fasting/refeeding treatments.
